# A community‐based dynamic choice model for HIV prevention improves PrEP and PEP coverage in rural Uganda and Kenya: a cluster randomized trial

**DOI:** 10.1002/jia2.26195

**Published:** 2023-12-06

**Authors:** Elijah R. Kakande, James Ayieko, Helen Sunday, Edith Biira, Marilyn Nyabuti, George Agengo, Jane Kabami, Colette Aoko, Hellen N. Atuhaire, Norton Sang, Asiphas Owaranganise, Janice Litunya, Erick W. Mugoma, Gabriel Chamie, James Peng, John Schrom, Melanie C. Bacon, Moses R. Kamya, Diane V. Havlir, Maya L. Petersen, Laura B. Balzer, for the SEARCH Study Team

**Affiliations:** ^1^ Infectious Diseases Research Collaboration Kampala Uganda; ^2^ Kenya Medical Research Institute Nairobi Kenya; ^3^ Department of Medicine Makerere University College of Health Sciences Kampala Uganda; ^4^ Global Programs for Research & Training Nairobi Kenya; ^5^ Division of HIV Infectious Diseases, and Global Medicine University of California San Francisco San Francisco California USA; ^6^ Department of Biostatistics University of Washington Seattle Washington USA; ^7^ Department of Health and Human Services National Institute of Health Bethesda Maryland USA; ^8^ Division of Biostatistics University of California Berkeley Berkeley California USA

**Keywords:** client‐centred, community health worker, dynamic choice, pre‐exposure prophylaxis, post‐exposure prophylaxis, village health team

## Abstract

**Introduction:**

Optimizing HIV prevention may require structured approaches for providing client‐centred choices as well as community‐based entry points and delivery. We evaluated the effect of a dynamic choice model for HIV prevention, delivered by community health workers (CHWs) with clinician support, on the use of biomedical prevention among persons at risk of HIV in rural East Africa.

**Methods:**

We conducted a cluster randomized trial among persons (≥15 years) with current or anticipated HIV risk in 16 villages in Uganda and Kenya (SEARCH; NCT04810650). The intervention was a client‐centred HIV prevention model, including (1) structured client choice of product (pre‐exposure prophylaxis [PrEP] or post‐exposure prophylaxis [PEP]), service location (clinic or out‐of‐clinic) and HIV testing modality (self‐test or rapid test), with the ability to switch over time; (2) a structured assessment of patient barriers and development of a personalized support plan; and (3) phone access to a clinician 24/7. The intervention was delivered by CHWs and supported by clinicians who oversaw PrEP and PEP initiation and monitoring. Participants in control villages were referred to local health facilities for HIV prevention services, delivered by Ministry of Health staff. The primary outcome was biomedical prevention coverage: a proportion of 48‐week follow‐up with self‐reported PrEP or PEP use.

**Results:**

From May to July 2021, we enrolled 429 people (212 intervention; 217 control): 57% women and 35% aged 15–24 years. Among intervention participants, 58% chose PrEP and 58% chose PEP at least once over follow‐up; self‐testing increased from 52% (baseline) to 71% (week 48); ≥98% chose out‐of‐facility service delivery. Among 413 (96%) participants with the primary outcome ascertained, average biomedical prevention coverage was 28.0% in the intervention versus 0.5% in the control: a difference of 27.5% (95% CI: 23.0–31.9%, *p*<0.001). Impact was larger during periods of self‐reported HIV risk: 36.6% coverage in intervention versus 0.9% in control, a difference of 35.7% (95% CI: 27.5–43.9, *p*<0.001). Intervention effects were seen across subgroups defined by sex, age group and alcohol use.

**Conclusions:**

A client‐centred dynamic choice HIV prevention intervention, including the option to switch between products and CHW‐based delivery in the community, increased biomedical prevention coverage by 27.5%. However, substantial person‐time at risk of HIV remained uncovered.

## 1 INTRODUCTION

Pre‐exposure prophylaxis (PrEP) and post‐exposure prophylaxis (PEP) are highly effective for HIV prevention [1, 2, 3] and have been recommended by the World Health Organization for persons at risk for HIV since 2014 [4]. In Eastern and Southern Africa, PrEP use increased from 400,000 persons in 2020 to 1,000,000 in 2021 [5]. However, PrEP uptake remains far short of the UNAIDS target of 2,000,000 persons by 2025 [5]; PEP use remains largely limited to occupational exposures [6], and HIV incidence declines have plateaued [5]. Barriers to using biomedical HIV prevention include stigma, distance to clinic, shifting risk perception, and rigid, clinic‐based screening and follow‐up [7, 8, 9, 10].

Delivery models that provide structured client‐centred choices, including the option to switch between biomedical prevention options (PrEP or PEP) over time, may help overcome these barriers. Several studies of hypothetical choices have been conducted to understand HIV prevention options that people at risk of HIV might prefer [11, 12, 13, 14], with heterogeneity in preferences for type, frequency and timing of PrEP [15] and HIV testing options [12, 16]. The marked variability in stated preferences underlines the reality that no single intervention is likely to meet the needs of all persons at risk for HIV, emphasizing the role of client‐centred, flexible models [17, 18].

Community‐based screening and prevention delivery offers an opportunity to provide client‐centred choices to persons who face barriers to accessing care at a health facility. However, community‐based delivery must leverage existing infrastructure to be scalable and sustainable. community health workers (CHWs) in Kenya and Uganda currently provide a range of primary healthcare services, including health promotion, management of common childhood illnesses, and follow‐up of expectant mothers and children aged ⩽5 years [19, 20]. Innovative CHW‐based delivery models have been shown to improve facility‐based delivery and newborn care [21] as well as the management of childhood illnesses [22]. To date, CHWs do not generally screen for HIV risk or deliver HIV prevention services.

We conducted a cluster randomized trial to test the hypothesis that a multi‐component intervention, offering structured client choices for HIV prevention product (PrEP or PEP), service location and testing, and delivered by CHWs with the support of clinicians would increase biomedical prevention coverage compared to referral for HIV prevention services at local health facilities.

## 2 METHODS

### 2.1 Setting and participants

Between May 2021 and August 2022, we conducted a cluster randomized study in 16 villages located in rural settings with high HIV prevalence in Southwestern Uganda (Ndeija) and Western Kenya (Homa Bay and Migori). We chose a cluster randomized trial design because the intervention was delivered in the village by CHWs, precluding individual randomization. Village inclusion criteria were location in a non‐adjacent geopolitical unit (i.e. a community with 9000–11,000 persons), served by a health facility providing antiretroviral therapy (ART), PrEP and PEP services, and with a community leader committed to study participation. Communities with ongoing HIV prevention interventions beyond country guidelines, in urban settings, and without a health centre providing ART, PrEP and PEP were excluded.

Residents of the 16 villages were eligible for participation if they were aged ≥15 years, HIV negative by country‐standard HIV testing algorithm and reporting current or anticipated HIV risk. HIV risk was assessed using in‐country Ministry of Health (MoH) screening instrument for PrEP initiation and two general questions regarding self‐assessed risk of HIV acquisition currently or in the next 3 months.

### 2.2 Study design and procedures

Prior to randomization, study villages were pair‐matched within community on village size, number of CHWs and proximity to a trading centre or highway. Randomization was conducted at a meeting of community leaders, where representatives from each matched pair selected and opened sealed envelopes to reveal the trial arm. Community leaders, CHWs, study staff and participants were not blinded to the randomization arm, but the study statistician (LBB) was blinded until trial completion.

In both intervention and control villages, an existing CHW who could speak English and conduct data collection using a phone app was selected for trial participation. In both arms, the selected CHW received training on routine HIV prevention, including key messaging about HIV testing, PrEP and PEP. For each CHW, 20 households under their care and with at least one person aged 15–39 years were randomly selected for study participation.

In all villages, CHWs conducted mobilization about study activities, including announcements at religious gatherings and community meetings. During enrolment, CHWs introduced study staff to the household head who provided verbal consent for household participation and general information about their household: number of members, their relationship to the household head and their age. Members of consenting households who were aged ≥15 years then provided individual written informed consent and were screened for eligibility (HIV negative by country‐standard testing algorithm, and with current or anticipated HIV risk).

In villages randomized to the control arm, participants reporting risk for HIV were referred to local health facilities for routine HIV prevention services. These services included confirmatory screening by an MoH health worker using standard tools, routine provision of PrEP or PEP if eligible and provision of HIV self‐test kits if requested and available. In villages randomized to the intervention, CHWs delivered the dynamic choice HIV prevention (DCP) model with clinician support, as detailed below. CHWs in both arms continued their existing MoH duties, such as the distribution of malaria bed nets and activities related to maternal‐child health. Table S1 provides a summary of the study arms and activities.

**Table 1:** Baseline characteristics of study participants, by arm and overall

	Intervention (*N* = 212)	Control (*N* = 217)	Total (*N* = 429)
**Women**	124/212 (58%)	119/217 (55%)	243/429 (57%)
**Aged 15–24 years**	76/212 (36%)	74/217 (34%)	150/429 (35%)
**Country**			
Kenyan	110/212 (52%)	100/217 (46%)	210/429 (49%)
Ugandan	102/212 (48%)	117/217 (54%)	219/429 (51%)
**Marital status**			
Single (never married)	64/212 (30%)	50/217 (23%)	114/429 (27%)
Married/cohabitating	134/212 (63%)	152/217 (70%)	286/429 (67%)
Divorced/separated/widowed	14/212 (7%)	15/217 (7%)	29/429 (7%)
**Occupation**			
Farmer	88/211 (42%)	80/217 (37%)	168/428 (39%)
Housewife	11/211 (5%)	19/217 (9%)	30/428 (7%)
Shopkeeper/Market vendor	17/211 (8%)	17/217 (8%)	34/428 (8%)
Student	37/211 (18%)	37/217 (17%)	74/428 (17%)
No job	7/211 (3%)	8/217 (4%)	15/428 (4%)
Manual labour/construction	11/211 (5%)	26/217 (12%)	37/428 (9%)
Fishing/Fishmonger	4/211 (2%)	1/217 (0%)	5/428 (1%)
Other	36/211 (17%)	29/217 (13%)	65/428 (15%)
**Risk enrolment criteria**			
Ministry of Health (MOH)	12/212 (6%)	23/217 (11%)	35/429 (8%)
Self‐assessed (current/anticipated)	47/212 (22%)	52/217 (24%)	99/429 (23%)
MOH and self‐assessed	153/212 (72%)	142/217 (65%)	295/429 (69%)
**HIV risk by sexual partners**			
Partner with HIV or unknown status (any, past 6 months)	119/212 (56%)	115/217 (53%)	234/429 (55%)
Primary partner with HIV^a^	12/159 (8%)	12/200 (6%)	24/359 (7%)
Primary partner with HIV on ART^b^	12/12 (100%)	10/12 (83%)	22/24 (92%)
**Alcohol use** (any, prior 3 months)	20/212 (9%)	20/217 (9%)	40/429 (9%)
**Pregnant** (women only)	11/122 (9%)	7/117 (6%)	18/239 (8%)
**Circumcised** (men only)	51/88 (58%)	52/97 (54%)	103/185 (56%)
**PrEP or PEP use** (any, prior 6 months)	5/212 (2%)	2/217 (1%)	7/429 (2%)

^a^ Among participants reporting a primary partner.

^b^ Among participants reporting their primary partner is with HIV.

### 2.3 Study intervention

Building on our experience offering PrEP and PEP in rural Kenya and Uganda [23, 24] as well as the growing body of literature on the importance of choice in HIV prevention [17, 18], we recognized that HIV risk, the need for biomedical prevention, barriers to use and preferences are dynamic over time and unique to each client. Therefore, our DCP multi‐component intervention was designed to empower clients with choices to individualize their own care. The DCP components, detailed below and in Figure S1, were informed by the PRECEDE framework [25, 26], and included: (1) community mobilization (*pre‐disposing*); (2) structured client choices with the option to switch over time (*enabling* and *reinforcing*; (3) phone access to a clinician 24/7 (*reinforcing*); and (4) clinician and CHW training on client‐centred care (*pre‐disposing, enabling and reinforcing*). Intervention development and implementation were also informed by feedback from key stakeholders at the national, regional and community levels.

**Figure 1:**
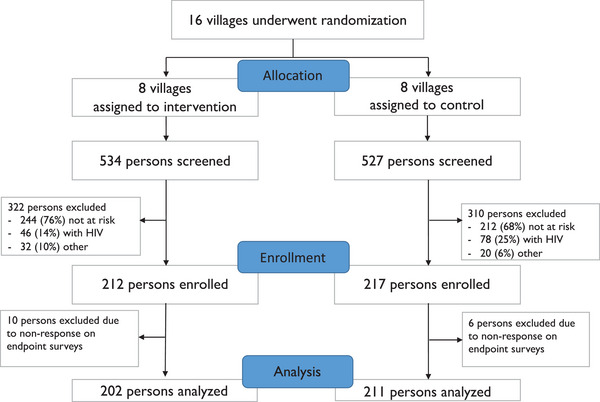
CONSORT diagram.

Prior to the study launch and every 6 months, both CHWs and clinicians received training on client‐centred care, including a framework for structured assessment of barriers and personalization of actions in response. Additionally, both CHWs and clinicians were trained on the DCP intervention and delivery, including structured choices by clients on HIV prevention services. CHWs were also trained on HIV testing, provided with job aids on HIV testing, and had monthly support supervision visits conducted by study laboratory personnel. Finally, external quality assurance and proficiency for HIV testing were performed with MoH panels, achieving 100% accuracy at both baseline and 6 months into the trial.

Intervention visits occurred at baseline and at weeks 4, 12, 24, 36 and 48 of follow‐up. Prior to study visits, additional mobilization was conducted by CHWs. At each visit, CHWs offered participants structured choices for HIV prevention services (Figure S1). Specifically, participants could choose between daily oral PrEP (TDF/XTC) or PEP (TDF/3TC/DTG). Clients selecting PrEP had the option of longer refills (3 months) compared to routine care (1 month, followed by 2 months, and then 3 monthly refills). Clients selecting PEP had an option of “pill‐in‐pocket,” where 3–5 pills were provided for immediate initiation following a future exposure. Post‐baseline product initiations or switches were facilitated by CHWs with clinician oversight and prescribing. All participants were seen in the community at baseline, but had the option to receive subsequent DCP services at the local clinic or offsite at a location of their choice. During follow‐up visits, participants also chose between HIV self‐testing or rapid testing. Structured barrier assessment to biomedical prevention and personalized plan development in response to client‐reported barriers also occurred at each visit. Participants were referred to counselling for psychological support due to trauma or gender‐based violence. Additionally, for reproductive health considerations or sexually transmitted infections, participants were referred to the local health facility, which offered service integration. Finally, all participants were provided a mobile hotline for 24/7 access to clinicians for general advice and questions as well as PrEP or PEP starts.

The DCP intervention was delivered by existing CHWs, leveraging their local knowledge and established relationships in the villages they serve. CHW delivery was overseen by clinicians, who supported rapid HIV testing and risk assessment, oversaw PrEP and PEP initiation, prescribed medications, facilitated switches between PrEP and PEP, responded to phone queries and facilitated refresher training for CHWs.

### 2.4 Study measures

In both arms, baseline demographics, HIV risk factors, and prior PrEP and PEP use were assessed at enrolment. For endpoint ascertainment at 24 and 48 weeks of follow‐up in both arms, CHWs and research staff conducted community‐based rapid HIV testing and HIV RNA testing (Gene Xpert, Cepheid) and administered a structured survey on PrEP and PEP use (i.e. pill ingestion) and HIV risk over the past 6 months. Specifically, for each month, participants were asked to retrospectively, self‐report general HIV risk, any risk due to sex with a partner with HIV or unknown status, any use of PrEP pills and any use of PEP.

### 2.5 Outcomes

The primary study outcome was biomedical prevention coverage, defined as the number of months during which a participant reported taking any PrEP or PEP pills divided by the number of months assessed. Biomedical prevention coverage during periods of self‐reported HIV risk was a pre‐specified secondary endpoint, compared between arms. We additionally report on HIV seroconversions, defined as any reactive HIV self‐test or HIV rapid test (per national testing algorithm) with confirmatory HIV RNA testing.

### 2.6 Statistical analysis

Accounting for the pair‐matched cluster randomized design [27], we estimated 16 villages (8 per arm) would provide 80% power to detect a difference of at least 15% in average biomedical prevention coverage, assuming 10% coverage in the control, a harmonic mean of 20 participants per cluster, a standard deviation of 0.35 and a matched pair coefficient of variation of k_m_ = 0.25. (See Statistical Analysis Plan [28] and CONSORT checklist in Supporting Information).

Primary and secondary outcomes were compared between arms with targeted minimum loss‐based estimation, accounting for the dependence of outcomes within clusters and adaptively adjusting for baseline covariates to maximize precision, while preserving Type‐I error control [29, 30]. Using the Student's *t‐*distribution, we calculated 95% confidence intervals (CIs) and tested the null hypothesis that the community‐based DCP intervention did not change HIV prevention coverage at the 5% significance level. Pre‐specified subgroups included sex, age group (15–24 years vs. 25+ years) and alcohol use (any vs. no use in 3 months prior to enrolment).

Within the intervention arm, we also summarized visit coverage and dynamic choice of the intervention components, including prevention product (PrEP, PEP, condoms only or none), location choice (out‐of‐clinic or clinic) and HIV testing (rapid or self‐testing) at baseline, week 24 and week 48.

### 2.7 Ethical approval

The Makerere University School of Medicine Research and Ethics Committee, the Uganda National Council for Science and Technology, the Kenya Scientific and Ethics Review Unit, the National Commission for Science, Technology and Innovation, and the University of California San Francisco Committee on Human Research reviewed and approved the study protocol. All participants provided written informed consent prior to study participation.

## 3 RESULTS

From May to August 2021, we enrolled 429 people (212 intervention; 217 control) in 16 villages in Southwestern Uganda and Western Kenya (Figure 1). Of these, 57% were women, 35% aged 15–24 years, 51% enrolled in Uganda, 67% married or cohabiting, 39% farmers and 17% students (Table 1). Participants were enrolled into the study based on MoH risk criteria (8%), current or anticipated self‐assessed HIV risk (23%) or both (69%). Alcohol use in the prior 3 months was reported by 9% of participants. Among women, 8% were pregnant; 56% of men were circumcised. Only 2% reported taking PrEP or PEP in the prior 6 months.

### 3.1 Intervention implementation

**Figure 2:**
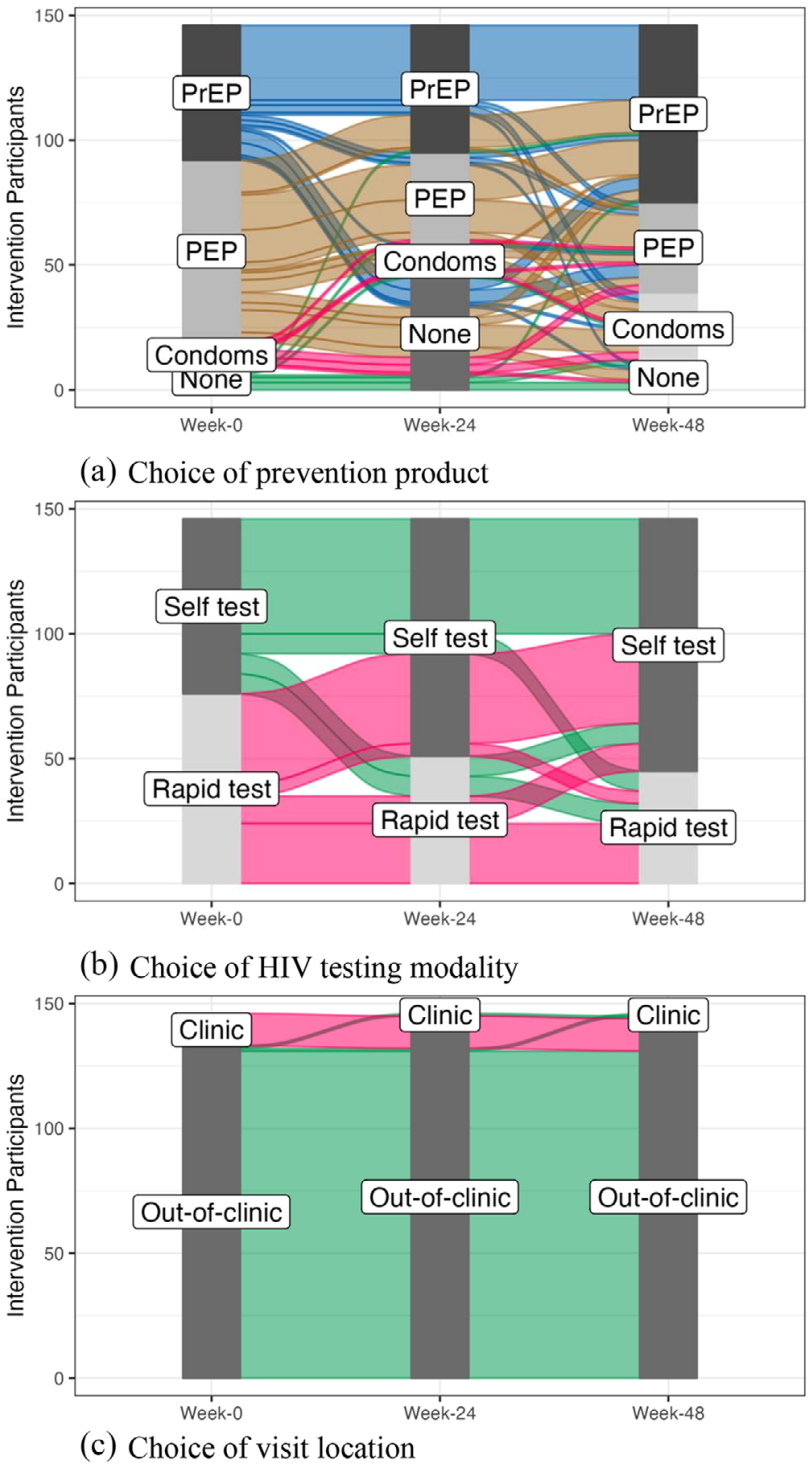
Choice of intervention components over time. (a) Choice of prevention product. (b) Choice of HIV testing modality. (c) Choice of visit location. Restricted to participants offered DCP at baseline, week 24 and week 48. (Plots including all intervention participants are given in Figure S3).

At scheduled visits, at least 80% of 212 intervention participants were seen by CHWs and offered the DCP package. Choice varied over time (Figure 2) and by sex (Figure S2). During the 48‐week follow‐up, 58% of intervention participants (60% of women and 56% of men) chose PrEP at least once, while 58% of participants (52% of women and 67% of men) chose PEP at least once. From baseline to week 48, selection of PrEP increased from 40% to 48%, while selection of PEP declined from 46% to 24%. Participants who chose self‐testing increased from 52% at baseline to 71% at week 48. Self‐testing was more popular among men (92% selecting at least once) than women (75% selecting at least once). At baseline, 93% of participants chose to have their follow‐up visits at an off‐site location rather than clinic; this increased to 99% by week 48; interest in off‐site visits was similar by sex.

### 3.2 Primary and secondary outcomes

The primary endpoint of biomedical prevention coverage, the proportion of follow‐up with self‐reported PrEP or PEP use, was ascertained in 413/429 (96%) participants: 202/212 (95%) of intervention participants and 211/217 (97%) of control participants. Average biomedical prevention coverage was 28.0% (95% CI: 23.5–32.4%) among intervention participants and 0.5% (95% CI: 0–1.0%) among control participants corresponding to an absolute increase of 27.5% (95% CI: 23.0–31.9%; *p* <0.001). Within the intervention arm, 14% of participants (28/202) were covered for 90% of follow‐up. In comparison, 98% of control participants (206/211) had no coverage during follow‐up.

**Figure 3:**
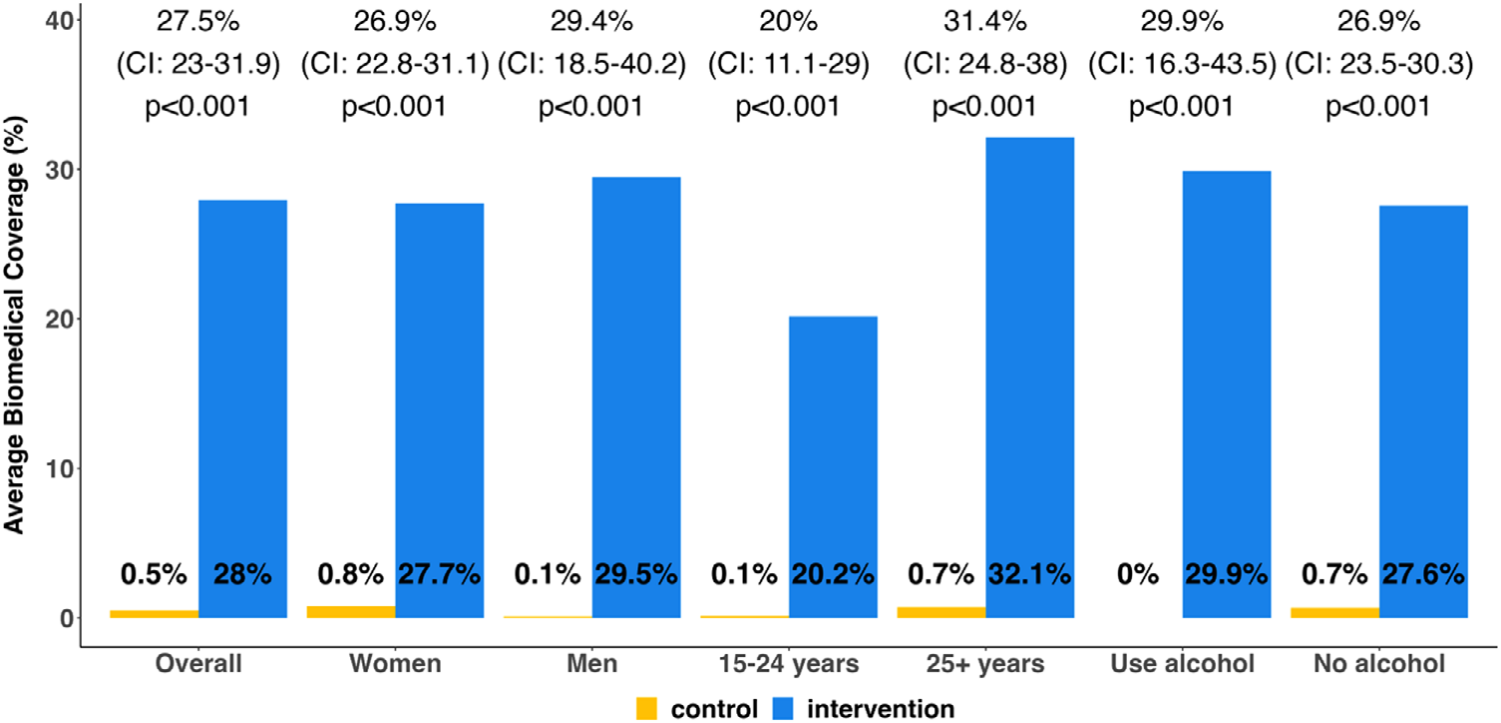
Effects on self‐reported use of biomedical prevention, overall and by subgroups. Effect estimates in terms of the difference in average biomedical prevention coverage between intervention and control arms.

The intervention significantly improved prevention coverage across key subgroups (Figure 3). Similar effect sizes were observed among women (26.9% increase), men (29.4% increase), persons who used alcohol (29.9% increase) and persons who did not use alcohol (26.9% increase). While coverage was lower among younger participants (15–24 years) than older participants (25+ years), the DCP intervention improved coverage in both groups: 20.0% increase and 31.4% increase, respectively.

**Figure 4:**
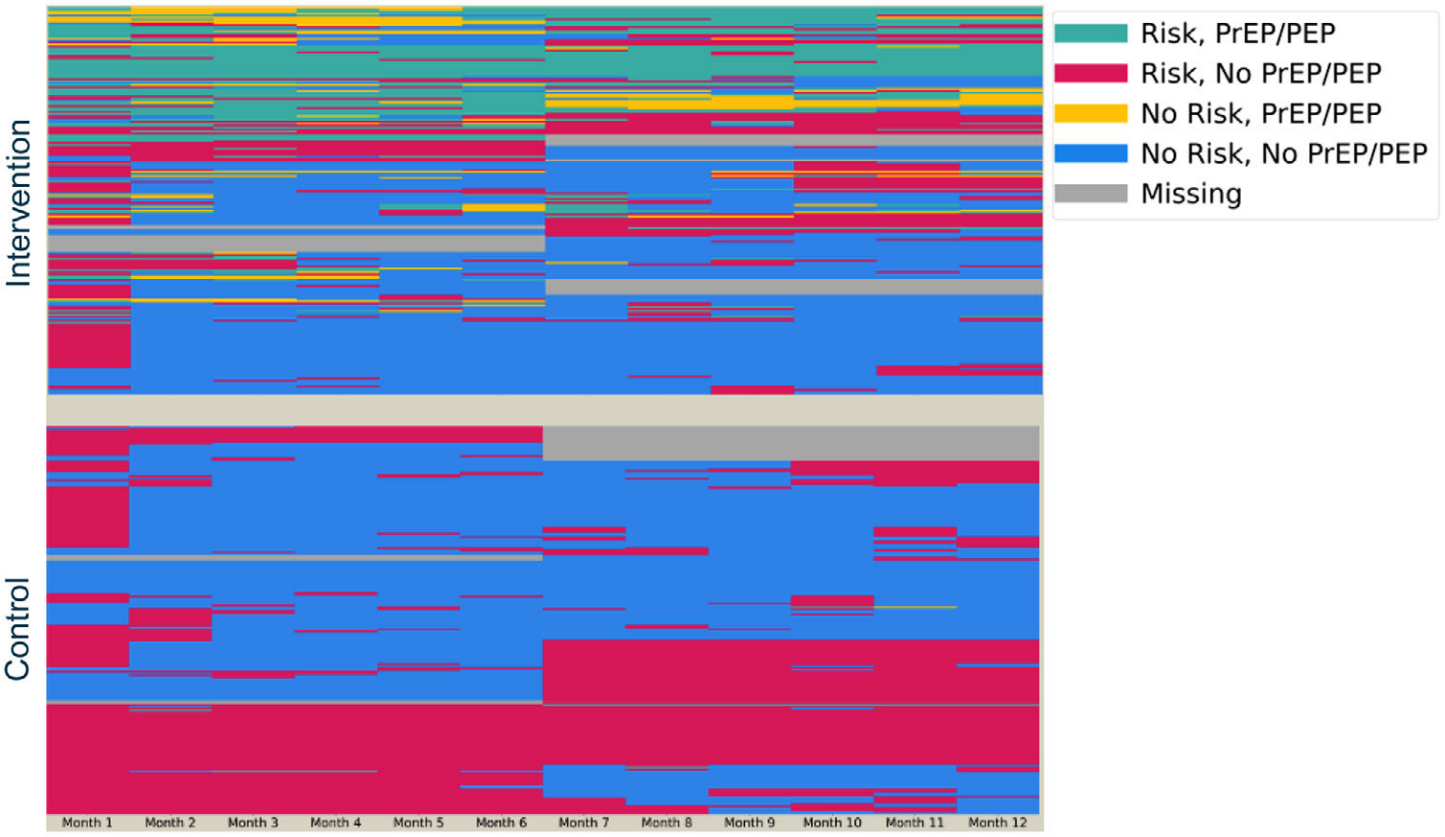
Heatmaps of self‐reported HIV risk and use of biomedical prevention, by arm over time. *Each row corresponds to a participant and each column a month of follow‐up*.

**Figure 5:**
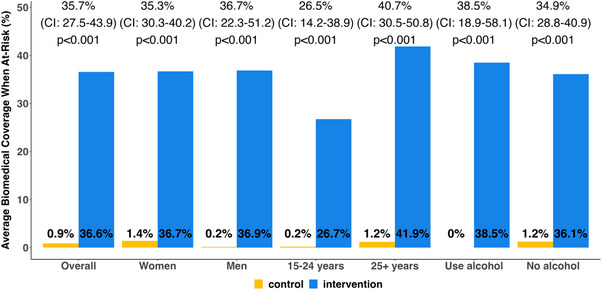
Effects on self‐reported use of biomedical prevention during periods of self‐reported HIV risk, overall and by subgroups. *Effect estimates in terms of the difference in average biomedical prevention coverage during periods of self‐reported HIV risk between intervention and control arms*.

Self‐reported HIV risk and use of biomedical prevention varied by arm and by time, as shown in Figure 4 where each row corresponds to a participant and each column to a month of follow‐up. Intervention effects were larger during periods of self‐reported HIV risk (Figure 5). Average prevention coverage was 36.6% among intervention participants versus 0.9% among control participants for an absolute increase of 35.7% (95% CI: 27.5–43.9, *p*<0.001). Again, similar effect sizes were observed for women (35.3%), men (36.7%), persons who used alcohol (38.5%) and persons who did not (34.9%). As before, intervention effects were larger among older adults (40.7%) than younger adults (26.5%).

Over the 48‐week follow‐up, two participants seroconverted. Both were in the intervention arm, and both were 24‐year‐old females. One had a partner of unknown status, and the other was in a polygamous marriage. Both chose PEP pill‐in‐pocket, but did not start PEP after unprotected sexual encounters.

## 4 DISCUSSION

In this cluster randomized trial, CHWs, supported by clinicians, delivered a community‐based, client‐centred dynamic choice model for prevention (DCP) to persons at risk of HIV acquisition. The DCP intervention provided clients with structured choices between biomedical prevention products (PrEP or PEP), service locations (clinic or out‐of‐clinic) and HIV testing options (self‐test or rapid test) as well as the ability to switch over time. A key innovation was bringing HIV prevention services into the community by leveraging existing CHWs. The intervention increased biomedical HIV prevention coverage by 27.5% overall and 35.7% during periods of self‐reported risk. Prevention coverage was <1% in the control, highlighting that offering PrEP or PEP does not equate to uptake.

Client preferences for HIV prevention have previously been explored in discrete choice experiments, providing insights on *stated* patient preferences for oral PrEP compared to injections [13], HIV self‐testing [12] and offsite location of HIV services [12]; however, the extent to which hypothetical preferences are realized in practice is less well‐understood. Further, both prior work and our study demonstrate significant heterogeneity in preferences, both across clients and over time, suggesting that a personalized approach is needed to optimize coverage and outcomes. However, specification and evaluation of prevention delivery models that offer structured client‐centred choices in real‐life settings and, in particular, models that integrate client choice in multiple dimensions (product, testing and location) remain sparse. In our DCP study, we presented participants with the opportunity to select oral PrEP or PEP based on individualized risk perception and preference and to switch between over time. We think this approach contributed to increased biomedical prevention coverage particularly during periods of risk.

HIV self‐testing was more popular than rapid testing in this study, and participant selection of self‐testing increased over time, highlighting the importance of this option in the community setting. Self‐testing has been widely accepted to be a safe alternative to rapid testing [31, 32, 33]. Additionally, participants in our study strongly preferred having their visits out‐of‐clinic. Indeed, only one participant chose to have their week‐24 visit at the clinic, and only one other participant chose to have their week‐48 visit at the clinic. Community‐based provision of HIV prevention services overcomes barriers to access, including transport costs, long waiting hours at the facility, stigma and lack of flexibility in mainstream, facility‐based HIV prevention models.

Our study increases the evidence of the potential for CHWs to extend HIV prevention services into the community. Previous literature has demonstrated the value of CHWs in improving other aspects of HIV care, specifically behavioural change counselling, health education, ART adherence counselling and defaulter tracing [34, 35, 36, 37] without decreasing the quality of care [38, 39]. Beyond HIV care, CHW‐led interventions have improved child health, specifically uncomplicated pneumonias, malaria and diarrhoea [40, 41], perinatal and neonatal outcomes [42, 43], and rapid diagnostic testing for malaria coupled with treatment of uncomplicated malaria [44, 45]. However, studies for CHW‐led delivery of HIV biomedical prevention are scarce. In a hyperendemic fishing community in the Rakai district of Uganda, CHW‐led PrEP counselling improved PrEP knowledge and use; however, in this study, PrEP delivery was clinic‐based, and no choices were offered [46]. In our study, CHWs in control villages referred clients for HIV prevention at local health clinics; thus, PrEP/PEP access was clinic‐based and delivered via standard approaches. In contrast, in intervention villages in our study, CHWs, supported by clinicians, offered and delivered HIV prevention services in the community. We believe our DCP intervention, its delivery and our results are generalizable to other rural settings with CHWs.

Prior studies have shown the effectiveness of CHWs in intervention delivery is impacted by various factors, including task‐specific training and capacity building [47], support and supervision to ensure continued motivation [48, 49, 50], a clear definition of roles [51] and community acceptance of their work [48]. In our pilot study, we provided training on client‐centred care and delivery of the DCP intervention to both CHWs and clinicians (Table S1). CHW roles were clearly defined and executed with clinician supervision throughout implementation. Finally, we engaged with community stakeholders during intervention development, and CHWs conducted multiple rounds of community mobilization.

Delivery of the DCP intervention by existing CHWs is potentially scalable, but additional research is necessary to understand effectiveness and implementation in routine practice. Since CHWs did not previously conduct HIV testing or PrEP/PEP delivery, clinician oversight was needed to ensure participant safety and intervention fidelity. In routine programmes, supervision will be required to ensure the safe delivery of HIV prevention services by CHWs but may not be as intensive as implemented in this pilot. We aim to address these and other open questions in a follow‐up, population‐level, community‐randomized trial (NCT05768763).

While the community‐based DCP intervention substantially increased biomedical prevention coverage, a significant proportion of time at risk remained uncovered. This could be explained, in part, by several barriers to taking PrEP within the household setting, including stigma and fatigue associated with daily pill taking. In addition, some participants were not comfortable disclosing their HIV risk status to CHWs and tensions within the household setting prohibited some from choosing oral PrEP or PEP. We provided location choice to address these barriers. In addition, the emergence of new biomedical products such as long‐acting injectable Cabotegravir could help to counter many of these barriers and further increase biomedical prevention coverage.

Our study has several limitations. First, we piloted a multi‐component intervention, which makes it challenging to parse out individual component effects. However, our results show both risk and choice change over time, suggesting the limitations of a one‐size‐fits‐all intervention and the importance of client‐centred service models. Open questions remain about the impact of each component of the intervention, as well as about its scale‐up. In particular, while choice was a core feature of our DCP model, our intervention also included other services (e.g. structured assessment of barriers). These other services, together with client‐centred choice, likely contributed to improvements in biomedical coverage, overall and during periods of risk. Ongoing qualitative and implementation science studies will help elucidate the specific impact of choice, and our follow‐up trial aims to assess scale‐up, implementation and impact of DCP at the population level. Second, our assessment of biomedical prevention coverage was by self‐report of any use of PrEP or PEP. As a result, covered time may have been over‐estimated; however, given the magnitude of the estimated effects, it is unlikely that bias due to self‐report or the survey limitations accounts for all, or even the majority, of the increases in biomedical coverage, seen overall and across key subgroups. Furthermore, 76% of participants reporting recent PrEP or PEP use had detectable tenofovir levels in their hair (Supporting Information). Third, our assessment of HIV risk is also subject to recall bias. Stigma or discomfort discussing HIV risk with CHWs may have led some participants to under‐report their retrospective HIV risk; however, the intervention effect on coverage was similar when evaluated for all follow‐up time (primary outcome) or restricted to follow‐up time at risk. Finally, assessing the cost of these interventions is important, and a costing study is ongoing.

## 5 CONCLUSIONS

In this cluster randomized trial, a CHW‐led, community‐based HIV prevention intervention that provided client‐centred dynamic choices in biomedical product, HIV testing and service location increased biomedical prevention coverage, both overall and during periods of risk, compared to community‐based referral for HIV prevention services at local health facilities. However, substantial time at risk of HIV remained uncovered, highlighting the need for additional interventions.

## COMPETING INTERESTS

DVH reports non‐financial support from ViiV Healthcare. All other authors declare no competing interests.

## AUTHORS’ CONTRIBUTIONS

ERK, JA, HS, EB, MN, GA, JK, CA, HNA, NS, AO, JL, EWM, GC, MRK, DVH, MLP and LBB conceptualized and designed the study. All authors participated in study operations or data collection. EWM, JP, JS and LBB performed the data analyses with input from ERK, JA, HS, EB, MN, GA, JK, CA, HNA, NS, AO, JL, GC, MCB, MRK, DVH and MLP. ERK, DVH, MLP and LBB drafted the manuscript with input from the other authors. All authors reviewed and approved the final manuscript.

## FUNDING

Research reported in this manuscript was supported by the U.S. National Institute of Allergy and Infectious Diseases (NIAID), the National Heart, Lung, and Blood Institute (NHLBI) and the National Institute of Mental Health (NIMH) and co‐funded under award number U01AI150510.

## DISCLAIMER

The content is solely the responsibility of the authors and does not necessarily represent the official views of the NIH.

## Supporting information


**Table S1**: Summary of study activities by trial arm
**Figure S1**: Schematic of the components of the dynamic choice HIV prevention (DCP) intervention, delivered by community health workers (CHWs) with clinician support.
**Figure S2**: Choice of intervention components over time by sex.
**Figure S2a**: Choice of prevention product by sex.
**Figure S2b**: Choice of HIV testing modality by sex.
**Figure S2c**: Choice of visit location by sex.
***(Restricted to participants offered DCP at baseline, week 24 and week 48)***.
**Figure S3**. Choice of intervention components among all intervention participants.
**Figure S3a**: Choice of prevention product.
**Figure S3b**: Choice of HIV testing modality.
**Figure S3c**: Choice of visit location.
**Supporting information about validation of self‐report with hair analyses**.
**Table S2**: CONSORT 2010 checklist of information to include when reporting a cluster randomized trial

## Data Availability

A complete de‐identified patient dataset sufficient to reproduce the study findings will be made available approximately 1 year after completion of the ongoing trial (NCT04810650), following approval of a concept sheet summarizing the analyses to be performed. Further inquiries can be directed to the SEARCH Scientific Committee at douglas.black@ucsf.edu.
