# Supplementary material for: A community‐based dynamic choice model for HIV prevention improves PrEP and PEP coverage in rural Uganda and Kenya: a cluster randomized trial
Source: J Int AIDS Soc. 2023 Dec 6;26(12):e26195. doi: 10.1002/jia2.26195 (PMC10698808; doi:10.1002/jia2.26195)
Supplement: Supplementary file 1 — Table S1: Summary of study activities by trial arm Figure S1: Schematic of the components of the dynamic choice HIV prevention (DCP) intervention, delivered by community health workers (CHWs) with clinician support. Figure S2: Choice of intervention components over time by sex. Figure S2a: Choice of prevention product by sex. Figure S2b: Choice of HIV testing modality by sex. Figure S2c: Choice of visit location by sex. (Restricted to participants offered DCP at baseline, week 24 and week 48) . Figure S3. Choice of intervention components among all intervention participants. Figure S3a: Choice of prevention product. Figure S3b: Choice of HIV testing modality. Figure S3c: Choice of visit location. Supporting information about validation of self‐report with hair analyses. Table S2: CONSORT 2010 checklist of information to include when reporting a cluster randomized trial [file JIA2-26-e26195-s001.docx]

**Supporting Table 1: Summary of study activities by trial arm.** Study visits among intervention participants occurred at least every 12 weeks (at baseline, week-4, 12, 24, 36, and 48). Study visits among control participants occurred at baseline, week-24, and week-48.

|  | **Intervention*** | **Control** |
| --- | --- | --- |
| CHW training on HIV prevention | X | X |
| Referral of clients to local health facilities for routine HIV prevention services by Ministry of Health staff |  | X |
| CHW and clinician training on and implementation of client-centered choice for biomedical prevention product: oral PrEP and PEP (including PEP pill-in-pocket) with the option to switch over time | X |  |
| Clinician training on and implementation of pill-in-pocket for PEP and of flexible refill duration for PrEP | X |  |
| CHW training on and implementation of client-centered choices for HIV testing: self-test or rapid test with the option to switch over time | X |  |
| CHW training on and implementation for client-centered choices for DCP delivery site: facility or out-of-facility  with the option to switch over time | X |  |
| CHW and clinician training on and implementation of client-centered care, including structured assessment of barriers to biomedical prevention and personalized plans in response | X |  |
| CHW training on and implementation of referrals to local health facilities for reproductive health, STIs, or psychological support | X |  |
| Clinician training on and implementation of mobile phone number access for client questions, available 24/7 | X |  |

*The DCP intervention, delivered by CHWs with clinician supervision, is shown in Supporting Figure 1.

**Supporting Figure 1: Schematic of the components of the Dynamic Choice HIV Prevention (DCP) intervention, delivered by community health workers (CHWs) with clinician support**

**
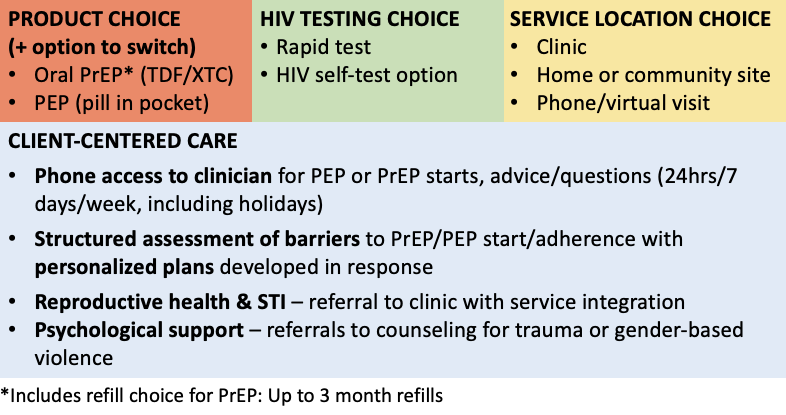
**

**Supporting Figure 2 – Choice of intervention components over time by sex.** Restricted to participants offered DCP at baseline, week-24, and week-48.


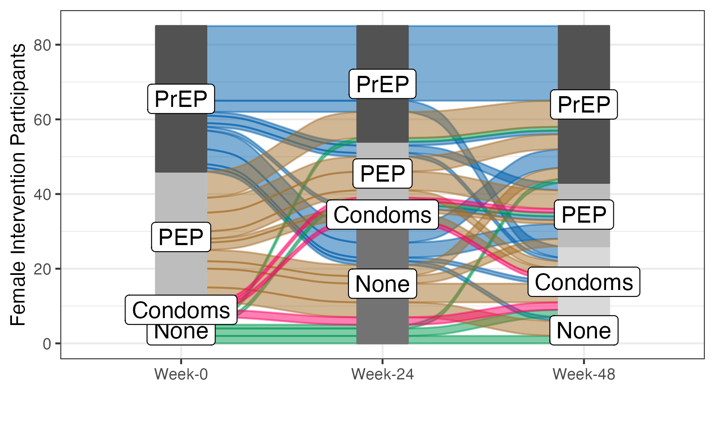

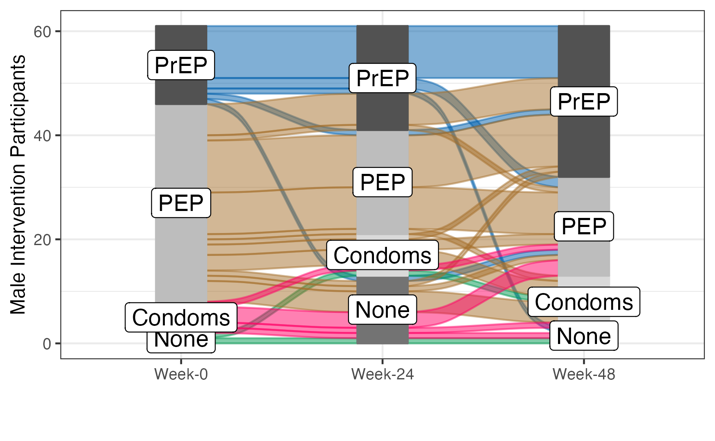


Supporting Figure 2a: Choice of prevention product by sex

**
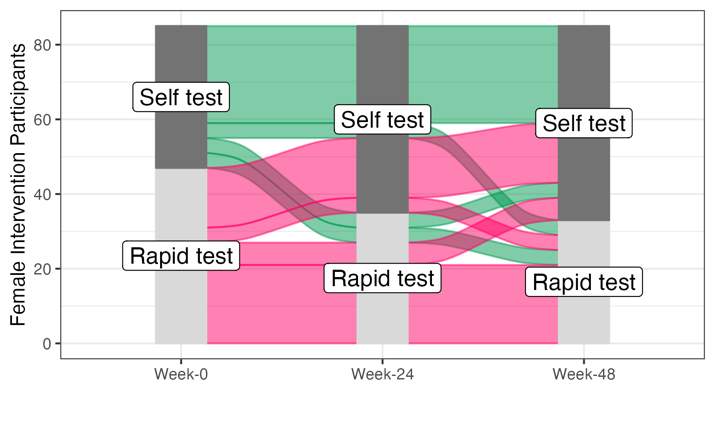

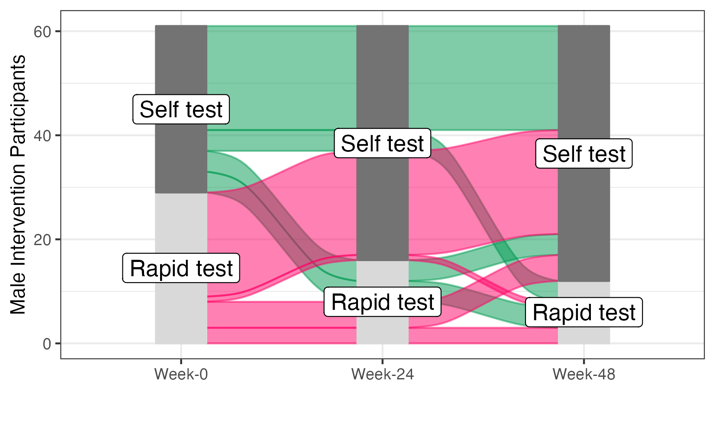
**

Supporting Figure 2b: Choice of HIV testing modality by sex


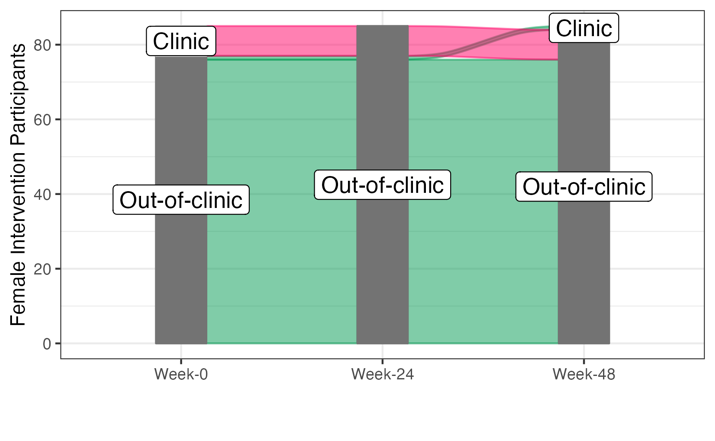

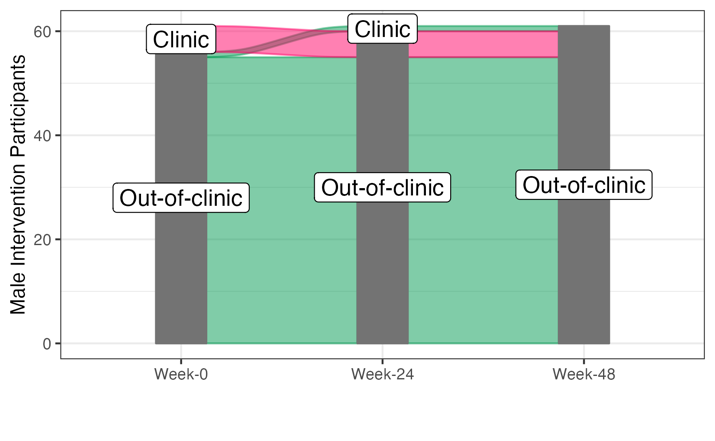


Supporting Figure 2c: Choice of visit location by sex

**Supporting Figure 3. Choice of intervention components among all intervention participants.** Choices are NA for participants who did not attend a given follow-up visit or did not reconsent to the ongoing extension study (NCT05549726) at week-48.


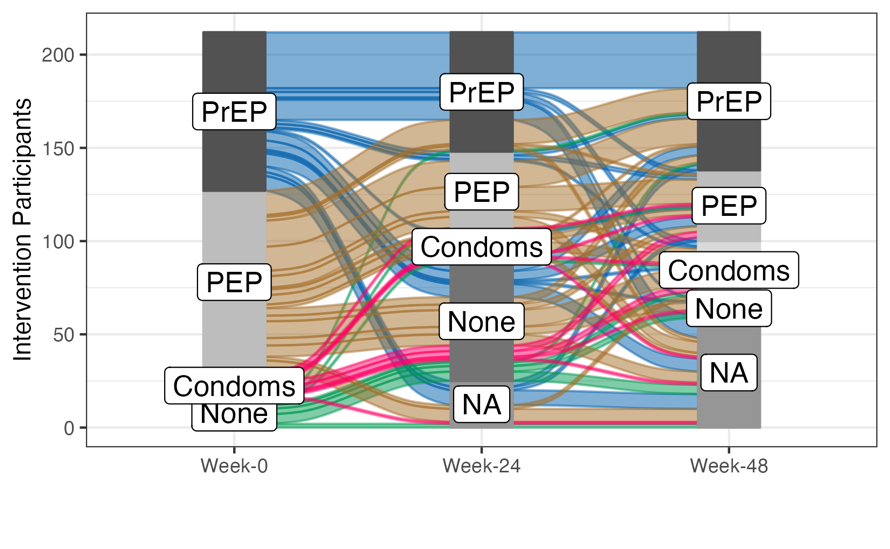


Supporting Figure 3a: Choice of prevention product


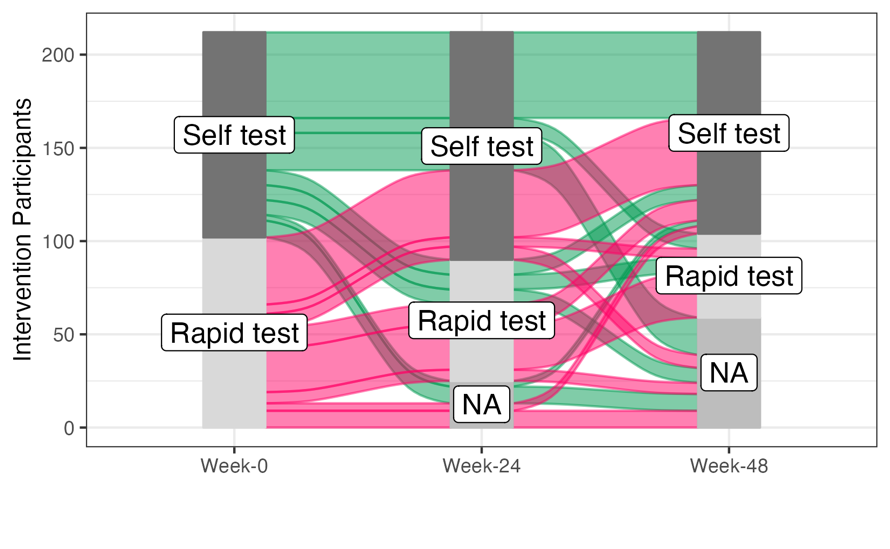


Supporting Figure 3b: Choice of HIV testing modality


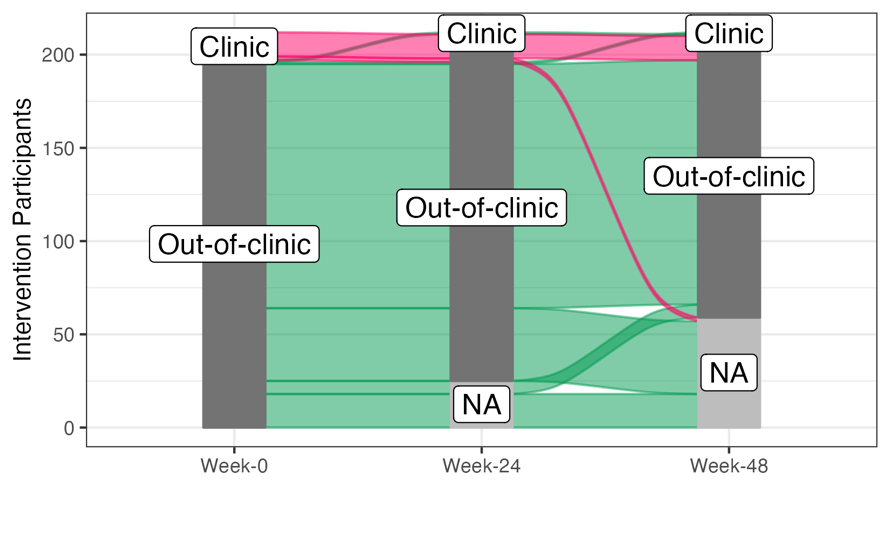


Supporting Figure 3c: Choice of visit location

**Supporting information about validation of self-report with hair analyses.**

Hair samples among participants reporting recent PrEP or PEP use at the week-24 survey were analyzed using chromatography-tandem mass spectrometry and validated methods in the Hair Analytical Laboratory at the University of California, San Francisco [1,2].

During the week-24 survey, 55 participants in the intervention arm and 0 participants in the control arm reported use of PrEP or PEP. Of the 29 intervention participants who reported recent use and had their hair sample analyzed, 22 (76%) had tenofovir concentrations corresponding to taking PrEP or PEP in the past 30 days.

[1] Liu AY, Yang Q, Huang Y, Bacchetti P, Anderson PL, Jin C, et al. Strong relationship between oral dose and tenofovir hair levels in a randomized trial: hair as a potential adherence measure for pre-exposure prophylaxis (PrEP). PloS one. 2014;9(1):e83736.

[2] Okochi H, Louie A, Phung N, Zhang K, Tallerico RM, Kuncze K, et al. Tenofovir and emtricitabine concentrations in hair are comparable between individuals on tenofovir disoproxil fumarate versus tenofovir alafenamide‐based ART. Drug testing and analysis. 2021;13(7):1354-70.

**Supplementary Table 2: CONSORT 2010 checklist of information to include when reporting a cluster randomised trial**

| Section/Topic | Item No | Standard Checklist item | Extension for cluster designs | Page No * |
| --- | --- | --- | --- | --- |
| Title and abstract | | | |  |
|  | 1a | Identification as a randomised trial in the title | Identification as a cluster randomised trial in the title | 1 |
|  | 1b | Structured summary of trial design, methods, results, and conclusions (for specific guidance see CONSORT for abstracts)^[[1]](#endnote-1),^^[[2]](#endnote-2)^ | See table 2 | Abstract (page 3-4) |
| Introduction | | | |  |
| Background and objectives | 2a | Scientific background and explanation of rationale | Rationale for using a cluster design | 6 |
|  | 2b | Specific objectives or hypotheses | Whether objectives pertain to the the cluster level, the individual participant level or both | 5 |
| Methods | | | |  |
| Trial design | 3a | Description of trial design (such as parallel, factorial) including allocation ratio | Definition of cluster and description of how the design features apply to the clusters | 6 |
|  | 3b | Important changes to methods after trial commencement (such as eligibility criteria), with reasons |  | N/A |
| Participants | 4a | Eligibility criteria for participants | Eligibility criteria for clusters | 6 |
|  | 4b | Settings and locations where the data were collected |  | 6 |
| Interventions | 5 | The interventions for each group with sufficient details to allow replication, including how and when they were actually administered | Whether interventions pertain to the cluster level, the individual participant level or both | 7-9; Supporting Table 1, Supporting Figure 1 |
| Outcomes | 6a | Completely defined pre-specified primary and secondary outcome measures, including how and when they were assessed | Whether outcome measures pertain to the cluster level, the individual participant level or both | 9-10 (details in SAP in the Supporting Info) |
|  | 6b | Any changes to trial outcomes after the trial commenced, with reasons |  | N/A |
| Sample size | 7a | How sample size was determined | Method of calculation, number of clusters(s) (and whether equal or unequal cluster sizes are assumed), cluster size, a coefficient of intracluster correlation (ICC or *k*), and an indication of its uncertainty | 9 (details in SAP in the Supporting Info) |
|  | 7b | When applicable, explanation of any interim analyses and stopping guidelines |  | N/A |
| Randomisation: | | | |  |
| Sequence generation | 8a | Method used to generate the random allocation sequence |  | 6 |
|  | 8b | Type of randomisation; details of any restriction (such as blocking and block size) | Details of stratification or matching if used | 6 |
| Allocation concealment mechanism | 9 | Mechanism used to implement the random allocation sequence (such as sequentially numbered containers), describing any steps taken to conceal the sequence until interventions were assigned | Specification that allocation was based on clusters rather than individuals and whether allocation concealment (if any) was at the cluster level, the individual participant level or both | 6 |
| Implementation | 10 | Who generated the random allocation sequence, who enrolled participants, and who assigned participants to interventions | Replace by 10a, 10b and 10c |  |
|  | 10a |  | Who generated the random allocation sequence, who enrolled clusters, and who assigned clusters to interventions | 6 |
|  | 10b |  | Mechanism by which individual participants were included in clusters for the purposes of the trial (such as complete enumeration, random sampling | 6-7 |
|  | 10c |  | From whom consent was sought (representatives of the cluster, or individual cluster members, or both), and whether consent was sought before or after randomisation | 7 |
|  |  |  |  |  |
| Blinding | 11a | If done, who was blinded after assignment to interventions (for example, participants, care providers, those assessing outcomes) and how |  | 6 |
|  | 11b | If relevant, description of the similarity of interventions |  | N/A |
| Statistical methods | 12a | Statistical methods used to compare groups for primary and secondary outcomes | How clustering was taken into account | 9-10 (details in SAP in the supporting info) |
|  | 12b | Methods for additional analyses, such as subgroup analyses and adjusted analyses |  | 9-10 (details in SAP in the supporting info) |
| Results | | | |  |
| Participant flow (a diagram is strongly recommended) | 13a | For each group, the numbers of participants who were randomly assigned, received intended treatment, and were analysed for the primary outcome | For each group, the numbers of clusters that were randomly assigned, received intended treatment, and were analysed for the primary outcome | Figure 1 |
|  | 13b | For each group, losses and exclusions after randomisation, together with reasons | For each group, losses and exclusions for both clusters and individual cluster members | Figure 1 |
| Recruitment | 14a | Dates defining the periods of recruitment and follow-up |  | 10 |
|  | 14b | Why the trial ended or was stopped |  | N/A |
| Baseline data | 15 | A table showing baseline demographic and clinical characteristics for each group | Baseline characteristics for the individual and cluster levels as applicable for each group | Table 1 |
| Numbers analysed | 16 | For each group, number of participants (denominator) included in each analysis and whether the analysis was by original assigned groups | For each group, number of clusters included in each analysis | 14; Figure 1 |
| Outcomes and estimation | 17a | For each primary and secondary outcome, results for each group, and the estimated effect size and its precision (such as 95% confidence interval) | Results at the individual or cluster level as applicable and a coefficient of intracluster correlation (ICC or k) for each primary outcome | 14-16; Figures 3-5 |
|  | 17b | For binary outcomes, presentation of both absolute and relative effect sizes is recommended |  | N/A |
| Ancillary analyses | 18 | Results of any other analyses performed, including subgroup analyses and adjusted analyses, distinguishing pre-specified from exploratory |  | 12-14, Figure 2, Supporting Figure 2 |
| Harms | 19 | All important harms or unintended effects in each group (for specific guidance see CONSORT for harms^[[3]](#endnote-3)^) |  | N/A |
| Discussion | | | |  |
| Limitations | 20 | Trial limitations, addressing sources of potential bias, imprecision, and, if relevant, multiplicity of analyses |  | 19 |
| Generalisability | 21 | Generalisability (external validity, applicability) of the trial findings | Generalisability to clusters and/or individual participants (as relevant) | 17-18 |
| Interpretation | 22 | Interpretation consistent with results, balancing benefits and harms, and considering other relevant evidence |  | 17-20 |
| Other information | | |  | Supplementary info |
| Registration | 23 | Registration number and name of trial registry |  | Abstract |
| Protocol | 24 | Where the full trial protocol can be accessed, if available |  | N/A |
| Funding | 25 | Sources of funding and other support (such as supply of drugs), role of funders |  | 20 |

1. Hopewell S, Clarke M, Moher D, Wager E, Middleton P, Altman DG, et al. CONSORT for reporting randomised trials in journal and conference abstracts. *Lancet* 2008, 371:281-283 [↑](#endnote-ref-1)
2. Hopewell S, Clarke M, Moher D, Wager E, Middleton P, Altman DG at al (2008) CONSORT for reporting randomized controlled trials in journal and conference abstracts: explanation and elaboration. *PLoS Med* 5(1): e20 [↑](#endnote-ref-2)
3. Ioannidis JP, Evans SJ, Gotzsche PC, O'Neill RT, Altman DG, Schulz K, Moher D. Better reporting of harms in randomized trials: an extension of the CONSORT statement. *Ann Intern Med* 2004; 141(10):781-788. [↑](#endnote-ref-3)
